# Environmental enrichment for neuropathic pain via modulation of neuroinflammation

**DOI:** 10.3389/fnmol.2025.1547647

**Published:** 2025-03-21

**Authors:** Jian-Dong Zhang, Zi-An Zhong, Wen-Yuan Xing

**Affiliations:** ^1^Physical Education College, Qilu Normal University, Jinan, China; ^2^Department of Sport Rehabilitation, Shanghai University of Sport, Shanghai, China

**Keywords:** environmental enrichment, neuropathic pain, neuroinflammation, cytokines, chemokines activating transcription factor 3, arginase-1, brain-derived neurotrophic factor, chronic constriction injury

## Abstract

Neuropathic pain causes tremendous biological and psychological suffering to patients worldwide. Environmental enrichment (EE) is a promising non-pharmacological strategy with high cost-effectiveness to reduce neuropathic pain and support rehabilitation therapy. Three researchers reviewed previous studies to determine the efficacy of EE for neuropathic pain to research how EE improves neuropathic pain through neuroinflammation. For this review, Embase, PubMed, and Cochran were searched. Three authors did study selection and data extraction. Out of 74 papers, 7 studies met the inclusion criteria. In the chronic constriction injury rats with acute or chronic detrimental stimulation, the change of pain behavior was influenced by environmental settings like start time, and cage size. Besides, physical EE has a larger effect than socially EE in inflammatory pain. These articles suggest employing various EE to regulate the release of pain-causing substances and changes in ion channels in the peripheral and central nerves to improve neuropathic pain behavior and depression and anxiety conditions. The existing proof provides important knowledge for upcoming preclinical investigations and the practical use of EE in clinical pain treatment. This analysis aids in the advancement of improved approaches for managing chronic pain, with a focus on internal mechanisms for controlling pain.

## 1 Introduction

The International Association for the Study of Pain defines neuropathic pain as “pain caused by a lesion or disease of primary afferent neurons in the somatosensory nervous system.” This type of pain is associated with profound neuroinflammation in the central nervous system (CNS) and peripheral nervous system (PNS) regions (Finnerup et al., 2021). Neuropathic pain is closely linked to the activation of immune cells and the release of inflammatory mediators (Fiore et al., 2023). Following nerve injury or disease, microglia in the CNS and macrophages in the PNS become activated and release pro-inflammatory cytokines, such as TNF-*α*, IL-1β, and IL-6 (Fiore et al., 2023; Bhol et al., 2024). These inflammatory mediators contribute to pain hypersensitivity by sensitizing nociceptive pathways in the spinal cord and peripheral neurons, leading to central sensitization and persistent pain states (Smith, 2023; Monteiro et al., 2020). Additionally, chemokines such as CCL2 and CX3CL1 facilitate neuroimmune interactions, further exacerbating neuroinflammation and chronic pain (Zheng et al., 2024). In some cases, severe neuroinflammation can promote the continued development of nerve pain, leading to chronic persistent pain. Recent studies have demonstrated that sustained glial activation and neuroinflammatory signaling drive the transition from acute to chronic neuropathic pain, highlighting the need for effective anti-inflammatory interventions (Song et al., 2024; Echeverria-Villalobos et al., 2023). According to the global epidemiological survey (Zhao et al., 2023), neuropathic pain affects 7 to 10% of the general population; it is a major threat to patients’ lives, health, and quality of life and has brought a huge economic and public health burden to society. Neuropathic pain is a complicated disease to treat (Xing et al., 2024). At present, the main treatment is drug therapy, such as using antidepressants, anticonvulsants, and opioids (Salvemini and Doyle, 2023). However, these drugs often have limited efficacy and significant side effects, making alternative therapeutic approaches essential (Alaoui Mdarhri et al., 2022).

Environmental enrichment (EE) is a preclinical model of rehabilitation used to facilitate voluntary motor, sensory, social, and cognitive activities by providing a stimulating environment and avoiding the risk of exercise overload (Tai et al., 2021). Evidence suggests that EE can alleviate neuropathic pain by modulating neuroimmune responses and reducing neuroinflammation (Kimura et al., 2019; Kimura et al., 2020; Wang et al., 2019; Wang et al., 2019). EE is achieved mainly by improving psychosocial factors, environmental novelty, physical aspects (increased activity and exercise), and sensitization of animal mechanical sensitivity threshold (Yam et al., 2018). Moreover, EE has been shown to suppress glial activation, reduce inflammatory cytokine levels (TNF-*α*, IL-1*β*, IL-6), and enhance anti-inflammatory signaling (IL-10, TGF-β) in both the CNS and PNS, leading to reduced pain hypersensitivity and improved functional recovery (Kimura et al., 2022).

Previous reviews on EE and neuropathic pain have shown that rodents exposed to EE exhibit reduced pain sensitivity compared to those in standard environments. However, a comprehensive review of the specific mechanisms and pathways EE modulates neuropathic pain and neuroinflammation has not yet been conducted. Therefore, this review focuses on summarizing the current understanding of how EE exerts its neuroprotective and anti-inflammatory effects in neuropathic pain conditions.

## 2 Materials and methods

### 2.1 Search strategy and inclusion criteria

Embase, PubMed, and Cochran were searched from December 1999 to December 2024 for relevant rat’s trials and neuropathic pain or neuroinflammation review. The following keywords are used as the primary targets in the search, either individually or jointly: T1 = “Environmental enrichment” OR “Enriched environment” OR “Swimming training” OR “Voluntary exercise” or “Wheel running” OR “Social activities.” T2 = “Neuroinflammation” OR “Neuropathic pain” OR “Neuroimmune” OR “Neurodegenerative dis-orders.” T3 = “Inflammatory factors” OR “Macrophage” OR “Inflammation factors” OR “Microglia.” When screening mice trials, the inclusion criteria are as follows: (1) Type of study. We included only published Randomized controlled trial articles that investigated the effects of EE on neuroinflammation and neuropathic pain in mice. The language of the article is limited to English; (2) Mice pain model. We included chronic constriction injury model mice, spinal nerve injury model mice and inflammatory pain model mice; (3) Interventions. When the mice are born or grow to a certain number of weeks, they will be divided into cages according to a certain number of cage breeding, intervention measures mainly include replacing larger cage, putting plastic cylinders, surgical caps, cardboard tubes, running wheels, tunnels of different styles and so on into the cage. After that, they change their toys every few days or weeks; (4) Types of outcome measures. The outcome measures mainly included mechanical sensitivity, temperature sensitivity, some of the experiments also measured depression, anxiety, motor capacity and inflammatory factors (for example TNF-*α*).

### 2.2 Study selection and data extraction

Three authors searched Embase, PubMed, and Cochran for all titles, abstracts, and text that matched the criteria, according to the screening criteria. For controversial papers, the two authors dis-cuss or let the corresponding author pass judgment. Papers that did not match the criteria for inclusion were omitted. The following information was extracted from the selected articles: (1) Published data (author, year); (2) Study design (mice pain model, sample size, control groups); (3) Intervention proto-col (including cage size, number of mice, type and number of toys, duration); (4) Outcome measures (mechanical stimulation, temperature stimulation, motor ability, etc.)

## 3 Efficacy of EE for neuropathic pain

Various preclinical studies have shown the clear effect of EE with different parameter settings (Table 1) for rodents with persistent neuropathic pain and inflammation. Pain models, including chronic constriction injury (CCI), spared nerve injury (SNI), and chronic compression of dorsal root ganglion, could reflect many facets of chronic pain in humans; in particular, hypersensitivity and presence of comorbidities, such as anxiety, depression, and cognitive deficit (Mouraux et al., 2021), have been investigated, and findings are translated into clinical pain management. The components of EE are physical enrichment (voluntary wheel running), social enrichment (group housing), and multifaceted stimulation through toy provision. A cage in different sizes is an indispensable physical component in EE and is more useful than regular-sized cages (26,656 cm^3^) and large cages (66,000 cm^3^) to improve pain behavior in neuropathic models (Kimura et al., 2019; Wang et al., 2019). The free-running wheel is one of the common physical environment modalities that facilitate the status of physical activity to promote exercise-induced hypoalgesia (Greenwood and Fleshner, 2019). Townsend et al. (2022) evaluated the effect of a 12-h voluntary running, 4 days a week for 5 weeks, on pain behavior. Sessions with high levels of exercise (within 3,300–9,600 m/day) before surgery demonstrated relief of ongoing pain in rats, but not in low runners (within 200–3,150 m/day). Compared with voluntary wheel running on the forced treadmill, high running with a peak rate of 16 m/min can block pain and reverse tactile hypersensitivity (Schmitt et al., 2020). Group housing consists of social enrichment. Stickney and Morgan (2021) conducted a trial and found that the social condition of as few as two rats could promote recovery by reducing pain and pain-evoked responses; however, the upper limit of the house group remains unknown. Toy provision could provide multifaceted stimulation in rat models of pain. Different combinations of climbing platforms, balls, tubes, cylinders, and caps have a positive effect on modifying chronic pain development (Kimura et al., 2019; Kimura et al., 2020; Wang et al., 2019; Wang et al., 2019; Chen et al., 2022).

**Table 1:** Environmental enrichment improves neuropathic pain in animal studies.

Study Author	Pain Model	Enrichment Environment Type	Standard Environment type	Environment duration	Indicators (Pain and inflammatory)	Key outcomes
Kimura et al. (2019)	CCI Rats	The cage (49*34*16 cm) Plastic cylinders, surgical caps, or cardboard tubes After 5 weeks, each object type changed weekly 5 per cage	Standard environment	7 WK	Threshold of paw contraction response to mechanical stimulation The latency of tail withdrawal after thermal stimulation	Pain and thermal pain behavior improved
Kimura et al. (2020)	CCI Rats	The cage (60*50*22 cm) Disposable caps, ping-pong balls, acrylic tubes, acrylic cabin, or acrylic rectangular housing 5 per cage	The cage (49*34*16 cm) with cardboard tubes, plastic cylinders, or disposable caps, and changed weekly	7 WK	Threshold of paw contraction response to mechanical stimulation	Pain behavior improved and anxiety behavior improved
Parent-Vachon and Vachon (2018)	SNI Rats	Ferret Home cage (31*20*41.5 inches) Colored balls, paper, PVC tubing, plastic spirals and so on 8 per cage	Standard environment (2 per cage)	Over 8 WK	Threshold of paw contraction response to mechanical stimulation	Pain behavior improved and anxiety behavior improved
Parent-Vachon et al. (2019)	SNI Mice	Ventilated polycarbonate cage Colored plastic hut, running wheel, and marbles 3 per cage	Single housing with only cotton nesting materials	8 WK	Threshold of paw contraction response to mechanical and cold stimulation,	Pain and cold pain behavior improved
Wang et al. (2019)	CCI Mice	Large polycarbonate cages (75*45*40 cm) Running wheels, toys, houses, and a maze-like tube system The configuration changed every 3 days 5 per cage	Standard environment	4 WK	Threshold of paw contraction response to mechanical stimulation	Pain behavior improved
Wang et al. (2019)	CCI Mice	Large polycarbonate cage (75*45*40 cm) Running wheels, toys, houses, and maze-like tube system. The configuration changed every 3 days 5 per cage	Standard environment	6 WK	Threshold of paw contraction response to mechanical stimulation; TNF-α	Pain and inflammatory behavior improved
Zheng et al. (2017)	Inflammatory pain model mice	Three-layered Plexiglas room Running wheels, tunnels of different styles, screw-type ladders, swings, and so on. 8 per cage	Standard environment	2 WK	Threshold of paw contraction response to mechanical stimulation	Pain behavior improved

CCI, Chronic constriction injury; SNI, Spinal nerve ligation; TNF-α, Tumor necrosis factor-α; WK, week.

EE with a variety of novel objects and large living spaces can weaken the reduction of pain threshold, depression-like phenotype, and memory deficits in mice with neuropathic pain after CCI (Wang et al., 2019). Kimura et al. (2019) established and tested two models of EE in a similar social environment; they found that compared with simple EE (sEE), in which enrichment started after weaning in regular-size cages with three different objections on regularly change (cardboard tubes, plastic cylinders, and disposable caps), pain behavior was reduced only by improved EE (iEE) condition, in which enrichment started at birth with five different objects weekly adding in order (like ping-pong balls, acrylic tubes, and cabin) in bigger cages (66,000 cm^3^). This research also argued that CCI rats could abolish acute and chronic neuropathic pain behavior after living under iEE conditions for 14 days; even after weaning, introducing sEE provided sufficient stimulation to reduce animal anxiety (Kimura et al., 2019). Current research using inflammatory pain models of mice found that living in EE 2 weeks with two running wheels, three different types of tunnels, a screw-type ladder, a swing, a teeterboard, a nest made of medical cotton, and a crib (8 per cage) improved pain behavior substantially (Estrázulas et al., 2022). Most studies reported that EE could improve neuropathic pain associated with physical activity (Wang et al., 2019; Leitzelar and Koltyn, 2021; Toloui et al., 2023); however, without sports environment, such as running wheels, social and inanimate interaction as analgesic and neuroimmune manipulator could prevent nerve degeneration upon CCI because exposure to EE impedes the development of pathological pain after chronic nerve injury (Kimura et al., 2020). A physically enriched environment (PE), such as living in large cages (±220,000 cm^3^) with various inanimate items, such as tunnels, running wheels, and extra nesting materials, has a larger effect on inflammatory pain than a socially enriched environment (SE), such as living in a group with four rats in the social environment and standard cages (±40,000 cm^3^) (Sadler et al., 2022). For psychological problems, EE (physically and socially enriched) can improve depression and anxiety-like behavior in the pain model of rats compared with the standard environment; nevertheless, no significant differences were found in SE, EE, and PE groups as well as in iEE and sEE groups (Kimura et al., 2020; Parent-Vachon and Vachon, 2018). In addition, many studies showed that EE can improve psychological problems, especially depression and anxiety-like behavior (Sparling et al., 2020; Ji and Xia, 2022; Smail et al., 2020).

## 4 Mechanisms of EE for neuropathic pain

**Figure 1:**
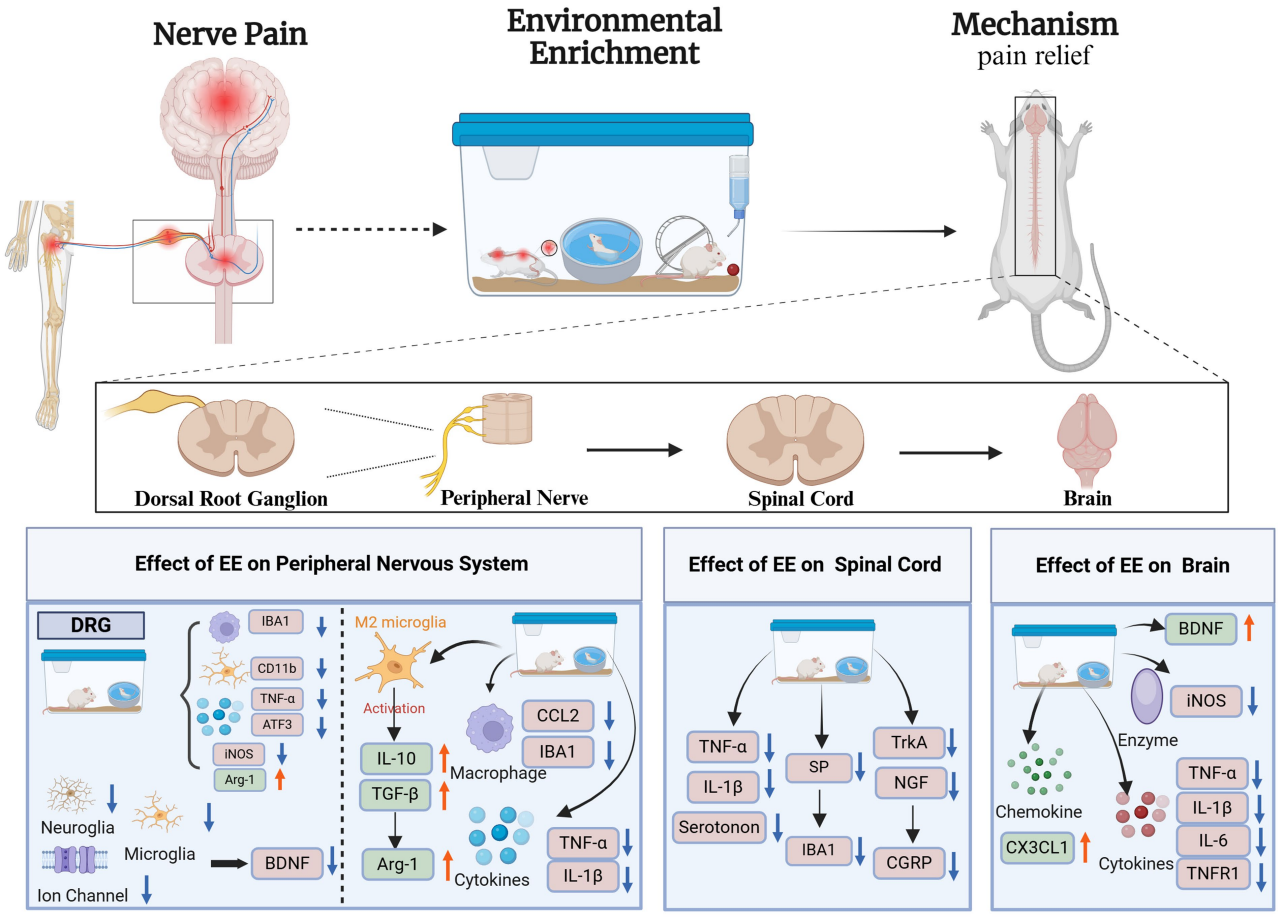
Mechanism of environmental enrichment therapy for neuropathic pain. Activating Transcription Factor 3 (ATF3); Arginase-1(Arg-1); Brain-Derived Neurotrophic Factor (BDNF); CX3C Chemokine Ligand 1 (CX3CL1); Chemokine Ligand 2 (CCL2); Calcitonin Gene-Related Peptide (CGRP); Cluster of Differentiation 11b (CD11b); Inducible Nitric Oxide Synthase (iNOS); Ionized Calcium Binding Adaptor Molecule 1 (IBA1); Interleukin-1β (IL-1β); Interleukin-6 (IL-6); Interleukin-10 (IL-10); Nerve Growth Factor (NGF); Substance P (SP); Tropomyosin Receptor Kinase A (TrkA); Tumor Necrosis Factor Receptor 1 (TNFR1); Tumor Necrosis Factor-α (TNF-α); Transforming Growth Factor-β (TGF-β); Dorsal Root Ganglion (DRG); Environmental Enrichment (EE). Created with BioRender.com.

According to anatomical studies, pain perception is caused by nociceptive neurons, which are activated in peripheral sensory nerves to produce nociceptive signals; the signals are then transmitted through dorsal root neurons and project onto the thalamus and the cerebral cortex (Figure 1) (Li et al., 2021; Chen et al., 2021). Neuropathic pain is associated with excessive inflammatory responses in the CNS and PNS (Fiore et al., 2023) and is caused by nerve damage (Ji et al., 2018). The neuropathic pain phenotype is characterized by the initiation and maintenance of the hypersensitivity of the CNS or PNS, neuroglia, activation of macrophages and white blood cells, recruitment of peripheral immune cells, and release of inflammatory mediators, such as cytokines (IL-1β, TNF-*α*, IL-6), chemokines (CCL2, CX3CL1), lipid mediators (PGE2, PGI2), and reactive oxygen species (Yu et al., 2020). These substances have the undesirable effect of sensitizing and irritating nociceptors, leading to persistent neuropathic pain (Hakim et al., 2024).

### 4.1 Effect of EE on the peripheral nervous system

The dorsal root ganglion (DRG) plays a crucial role in pain transmission by relaying nociceptive signals to the spinal cord (Jang and Garraway, 2024). When peripheral nerves are injured, white blood cells, macrophages, and neuroglia in DRG are involved in immune responses, leading to pain (Rios et al., 2021; Msheik et al., 2022). EE modulates neuroinflammation in the PNS primarily by regulating immune cell activity and inflammatory signaling pathways.

The macrophage chemokine CCL2 is associated with neuropathic pain and inflammation in DRG. CCL2 consistently induces mechano-nociceptive hypersensitivity (Dansereau et al., 2021) and activates microglia after peripheral nerve injury (Popiolek-Barczyk et al., 2020). Gu et al. (2021) found that EE can reduce the expression of inflammatory factors, such as macrophage marker IBA1, microglia CD11b, and chemokines CCL2 and ATF3 (a transcription factor considered as a marker of nerve damage) in DRG to achieve pain relief. Another study (Kimura et al., 2020) found that DRG attenuated the expression of ATF3 and iNOS and increased the expression of the anti-inflammatory marker Arg-1 in CCI injury after EE. Xu et al. (2025) studied chronic neuropathic pain in rats and found the enrichment of proteins related to “antioxidant activity” in ganglia. These proteins are involved in analgesia and act on protein-coupled receptors, causing the inactivation of calcium channels to reduce pain.

Peripheral tissue or nerve injury increases the expression of BDNF in DRG (Smith, 2024). Wu et al. (2021) reported that BDNF at the level of DRG can contribute to pain transmission as well as pain hypersensitivity and central sensitization mainly because BDNF can enhance the excitability of the dorsal horn of the spinal cord and promote the transmission of pain after nerve injury mainly by regulating glutamatergic excitability and GABAergic/glycinergic to inhibit nerve conduction. Previous studies found (Thakkar and Acevedo, 2023; Huang et al., 2021) that BDNF can act as a factor that contributes to the onset and development of pain and a pain mediator that regulates pain. Thus, the release of BDNF in DRG is undesirable because it contributes to the development of nerve pain. A study reported that EE can reduce the expression of BDNF in DRG by reducing neuroglia activation, chemokine and cytokine expression, and ion channel activity to alleviate hyperalgesia after partial nerve injury to inhibit neuropathic pain (Tian et al., 2018).

Immune cells are related to the occurrence of inflammation after peripheral nerve injury (Vergne-Salle and Bertin, 2021). Inflammation drives microglia and inflammatory cells to release several proinflammatory cytokines and chemokines (such as TNF-*α*, IL-1*β*, CCL2, and CCL3). TNF-α is a key proinflammatory cytokine that binds to the receptor TNFR1 to activate the signal pathways of NF-κB and p38 MAPK and trigger inflammatory responses (Duan et al., 2022). One study found (Chu et al., 2020) that EE reduces TNF-*α* and IL-1β levels in the sciatic nerve, hinders the activation of inflammatory signaling pathways, and relieves peripheral neuropathic pain. Wallerian degeneration occurs after peripheral nerve injury and is an important degenerative process that occurs at the distal stump of the nerve in response to nerve fiber damage (Tian et al., 2025). Kimura et al. (2020) found that an EE could reduce the occurrence of Wallerian degeneration by significantly retaining myelinated nerve fibers in the proximal part of the sciatic nerve with CCI, thereby affecting the reduction of nerve pain in sciatic nerves. In another study (Kimura et al., 2020), researchers found that EE can promote the expression of Arg-1, an enzyme that initiates downstream pathways associated with cell proliferation and tissue repair. As such, changes in Wallerian degeneration might be related to Arg-1. Yu et al. (2020) found increased Arg-1 expression in the sciatic nerve after EE and decreased expression of the macrophage markers Iba-1 and CCL2 while significantly ameliorating neuropathic pain in CCI mice. Microglia activation is mainly divided into M1 and M2. The activation of M1 is a pro-inflammatory state, which releases cytokines and chemokines, while the activation of M2 is an anti-inflammatory state, which releases Arg-1 and IL-10. M2 activation promotes the release of anti-inflammatory cytokines, such as IL-10 and TGF-*β*, and induces Arg-1, which promotes the conversion of arginine into polyamines to ease the onset of inflammation (Arabpour et al., 2021).

In the peripheral nervous system, one major pathway involved is the NF-κB signaling pathway, which is crucial for the production of pro-inflammatory cytokines such as TNF-*α* and IL-1β (Guo et al., 2024). Peripheral nerve injury leads to NF-κB activation through the phosphorylation and degradation of IκB*α*, allowing p65/p50 nuclear translocation and subsequent transcription of inflammatory mediators (Giridharan and Srinivasan, 2018). Studies have shown that EE downregulates NF-κB activation in DRG by inhibiting the phosphorylation of IKK complex, reducing TNF-α and IL-1β expression (Wu et al., 2020). Additionally, the MAPK signaling pathway is another critical target of EE in the PNS. The activation of p38 MAPK in response to nerve injury enhances pro-inflammatory cytokine release, leading to nociceptor sensitization (Falcicchia et al., 2020). EE has been demonstrated to suppress p38 MAPK phosphorylation, thereby attenuating the inflammatory cascade in DRG neurons and associated macrophages (Martina et al., 2023). Furthermore, EE influences the JAK/STAT pathway, particularly by downregulating STAT3 phosphorylation, which is known to promote IL-6 expression and neuroinflammation (Lv et al., 2024). Another essential mechanism of EE in the PNS is its regulation of immune cell polarization (Udit et al., 2022). EE exposure has been linked to increased expression of Arg-1, a marker of M2 anti-inflammatory macrophages while reducing IBA-1 expression, which is associated with pro-inflammatory M1 macrophages (Assis et al., 2022). This shift toward an M2-dominant phenotype supports nerve regeneration and pain relief by enhancing anti-inflammatory cytokine production, such as IL-10 and TGF-β (Liang et al., 2024).

### 4.2 Effect of EE on the spinal cord

Pain signals from peripheral nerves are integrated and modulated in the spinal cord before being transmitted to higher brain regions (Gurba et al., 2022). CNS sensitization is caused by the abnormal activation of ion channels, spinal microglia, and astrocytes and by the release of pain signals, such as inflammatory cytokines, SP, and CGRP (Cui et al., 2023). EE plays a crucial role in mitigating central sensitization by regulating inflammatory signaling within microglia and astrocytes.

Gong et al. (2018) found that EE can reduce pain and decrease the release of IL-1β and TNF-*α*. SP neuropeptide and calcitonin gene-related peptides are important pain mediators released by nociceptive sensory nerve endings, which promote central nervous hypersensitivity mainly through the release of inflammatory cytokines and chemokines through MRGPRX2 activation (Green et al., 2019). He et al., 2022) found that SNI mice exposed to EE had significantly lower levels of SP and calcitonin genes in the spinal cord thereby improving pain in SNI mice. Another study (Hsieh et al., 2022) found that EE significantly reduced SP expression, improved microglial hyperactivation, and reduced IBA1 expression to restrain pain. For the calcitonin gene-related peptide, the EE may inhibit the release of TrkA or NGF to decrease the sprouting of CGRP+ fibers, which may be the reason for the reduction of calcitonin gene under EE (Sliwinski et al., 2018). During chronic pain, a decrease in serotonin from the rostral ventromedial medulla to the spinal cord helps to reduce pain. Wang et al. (Takeura et al., 2019) detected decreased serotonin and microglia macrophage markers in the spinal cord after EE in CCL pain model mice. Tai et al. (2021) found that using ketamine in combination with EE could improve spinal cord injury (SCI) pain models, decrease astrocytes, and suppress neurons and microglia NR2B subunits of NMDAR; the activation of NMDAR and NR2B is particularly associated with nerve pain at the lesion site of the spine. The activation of NF-κB and interleukin (IL -1β) is enhanced, resulting in hyperalgesia (Wang et al., 2022). The combination of ketamine and EE reduces inflammatory processes, such as interleukin (IL-1β) in the spinal cord, thereby reducing nerve pain and inflammation; this finding provides a novel scheme for the intervention of nerve pain in the future. In general, EE mainly reverses mechanical allergy in the spinal cord, reduces pro-inflammatory factors in nerve pain, increases anti-inflammatory substances in the spinal cord, and inhibits microglia and astrocyte activation to relieve pain, particularly CNS inflammation after SNI and SCI.

In the spinal cord, a key mechanism is the inhibition of NF-κB and p38 MAPK pathways, which are highly active following nerve injury and contribute to chronic pain states (Guo et al., 2024). EE reduces TNF-*α* and IL-1β expression in the spinal cord by downregulating NF-κB nuclear translocation and suppressing p38 MAPK activation in spinal microglia (Su et al., 2021). Furthermore, EE modulates the JAK/STAT pathway, particularly through the inhibition of STAT3 phosphorylation, which has been implicated in astrocyte-mediated inflammation and neuroimmune interactions (Lv et al., 2024). Differently, EE differentially affects IBA-1 expression in the spinal cord compared to the PNS (Strath et al., 2021). While EE significantly downregulates IBA-1-positive microglia in the dorsal horn, it also reduces astrocyte activation, indicated by decreased GFAP expression (Han et al., 2024). This suggests that EE exerts broader anti-inflammatory effects in the spinal cord by targeting both microglia and astrocytes, whereas in the PNS, its primary action is on macrophage polarization (Liao et al., 2024). Moreover, EE modulates neurotransmitter signaling to counteract central sensitization (Wang et al., 2024). It increases GABAergic inhibitory transmission while reducing excessive glutamate release, thus balancing excitatory and inhibitory inputs in the spinal cord (Welle and Smith, 2025). Exposure to EE has been shown to enhance CX3CL1 (fractalkine) expression, which promotes microglial homeostasis and reduces neuroinflammation (Subbarayan et al., 2022; Angelopoulou et al., 2020).

### 4.3 Effect of EE on the brain

The brain plays a central role in processing and modulating pain perception. EE exerts significant neuroprotective effects in the thalamus, cerebral cortex, and hippocampus, primarily by reducing neuroinflammation and oxidative stress (Usrey and Sherman, 2023).

When inflammation occurs in the brain, it can induce oxidative stress and release pro-inflammatory factors, such as IL-1*β*, TNF-*α*, and NO (Bai et al., 2023). BDNF is a major CNS factor that regulates many different cellular processes by binding and activating TrkB, which is involved in the development and maintenance of normal brain function (Colucci-D'Amato et al., 2020). Neuropathic pain reduces the complexity of excitatory synapse junctions and BDNF expression in the hippocampus; this finding is due to the signal augment and microglia activation and the release of TNF-α and the receptor TNFR1 after neuropathic pain, leading to the dysregulation of the BDNF/TrkB pathway (Tao et al., 2022). Reducing the expression of pro-inflammatory cytokines in the brain is key to improving neuroinflammation and analgesia (Bhol et al., 2024; Karavis et al., 2023). Kalb et al. (2021) and Qian and Huang (2022) found that the EE attenuated the expression of IL-1 β and TNF-*α*, regulated the levels of BDNF in the CNS, and increased the expression of CX3CL1, which is a chemokine constitutively expressing in the brain and playing immunomodulatory and anti-inflammatory roles during acute and chronic neuroinflammation. Wang et al. (2019) demonstrated that EE attenuates TNF-α levels in the hippocampus, thereby reducing neuroinflammation in the hippocampus and reducing the pain threshold in mice with neuropathic pain after CCI. Camuso et al. (2022) also demonstrated that BDNF levels in the hippocampus and frontal cortex were significantly increased, and neuropathic pain was improved in mice exposed to 8 weeks of EE stimulation. Lee et al. (2023) found that long-term, EE stimulation reduced astrocyte and microglia activation in mice with neuropathic pain; the expression levels of TNF-α, IL-1β, IL-6, and iNOS in the brain of mice were becoming normal (NO is a highly unstable free radical gas associated with neurosensitization; iNOS is an enzyme produced by NO (Tewari et al., 2021). In general, EEs improve inflammation and pain by reducing the expression of pro-inflammatory factors and increasing the levels of BDNF.

One primary target of EE is BDNF/TrkB signaling, which is crucial for neuroplasticity and neuroimmune regulation (Cappoli et al., 2020). Neuropathic pain reduces BDNF levels in the hippocampus due to chronic inflammation and microglia activation (Lv et al., 2024). EE reverses this effect by upregulating BDNF expression, thereby restoring synaptic function and reducing pain-related emotional disturbances (Li et al., 2025). Furthermore, EE suppresses NF-κB-mediated inflammatory pathways in the hippocampus and cortex, reducing levels of TNF-*α*, IL-1*β*, and nitric oxide synthase (iNOS) (Gu et al., 2021). These effects help alleviate neuroinflammation and improve cognitive function in chronic pain states. Unlike in the PNS and spinal cord, where EE mainly affects microglia and macrophages, its effects in the brain extend to astrocytes (Bennett and Bennett, 2020). EE reduces GFAP expression, indicating decreased astrocyte reactivity, which is crucial for long-term pain modulation (Enbar et al., 2024). Another distinguishing feature of EE’s action in the brain is its impact on oxidative stress pathways (Ramos et al., 2024). EE enhances the expression of antioxidant enzymes, such as superoxide dismutase and catalase, which mitigate oxidative stress-induced neuronal damage in neuropathic pain conditions (Kapoor et al., 2019). Additionally, EE upregulates CX3CL1 signaling, which further suppresses microglial activation and promotes anti-inflammatory responses (Subbarayan et al., 2022).

## 5 Conclusion

This review summarizes the potential mechanisms by which EE can alleviate neuropathic pain via modulation of neuroinflammation. EE can relieve neuropathic pain by reducing inflammatory mediators, such as proinflammatory cytokines (IL-1β, TNF-α, IL-6) and chemokines (CCL2, CCL3), among others, increasing anti-inflammatory substances (IL-10, Arg-1, CX3CL1) in the periphery and CNS. Specifically, BDNF has an active role in the brain, but the opposite is true in the dorsal root ganglion. Although many studies found that EE can alleviate neuropathic pain, there is still a long way to understand the full mechanism. EE can be used as a non-pharmacological tool to ameliorate pain, anxiety, and depression in neurorehabilitation programs. A more in-depth investigation is required to optimize the integration of EE interventions with other medical approaches to enhance treatment outcomes.

## Glossary

ATF3: activating transcription factor 3
Arg-1: arginase-1
BDNF: brain-derived neurotrophic factor
CCI: chronic constriction injury
CX3CL1: CX3C chemokine ligand 1
CCL2: chemokine ligand 2
CCL3: chemokine ligand 3
CGRP: calcitonin gene-related peptide
CD11b: cluster of differentiation 11b
iNOS: inducible nitric oxide synthase
IBA1: ionized calcium binding adaptor molecule 1
IL-1β: interleukin-1β
IL-6: interleukin-6
IL-10: interleukin-10
NGF: nerve growth factor
SP: substance P
TrkA: tropomyosin receptor kinase A
TrkB: tropomyosin receptor kinase B
TNFR1: tumor necrosis factor receptor 1
TGF-β: transforming growth factor-β
TNF-α: tumor necrosis factor-α
PGI2: prostacyclin I2
PGE2: prostaglandin E2
NF-κB: nuclear factor kappa-B
MAPK: mitogen-activated protein kinase
MRGPRX2: Mas-related G-protein coupled receptor member X2
NMDA: *N*-methyl-D-aspartic acid
NO: nitric oxide
JAK/STAT: Janus kinase/signal transducer and activator of transcription
